# The Role and Mechanism of Nrf2 in Ameliorating Oxidative Stress and Inflammation in IR Mice by Aerobic Exercise

**DOI:** 10.3390/ijms27104310

**Published:** 2026-05-12

**Authors:** Xuan Liu, Yuqing Ding, Tao Chen, Zhengkang Wu, Shujuan Hu, Xianwang Wang

**Affiliations:** 1 School of Education and Physical Education, Yangtze University, Jingzhou 434023, China; 18235976373@163.com (X.L.);; 2 Department of Biochemistry and Molecular Biology, Center for Molecular Medicine, Yangtze University, Jingzhou 434023, China

**Keywords:** Nrf2, aerobic exercise, insulin resistance, oxidative stress, inflammation

## Abstract

This study explored the regulatory role of nuclear factor E2-related factor 2 (Nrf2) in aerobic exercise improving oxidative stress and inflammatory responses in mice with insulin resistance (IR) induced by a high-fat diet. We established an IR mouse model through a high-fat diet, then subjected the IR mice to aerobic exercise, intraperitoneal injection of luteolin, or a combined intervention. After 6 weeks of intervention, we measured serum lipid and glucose profiles; evaluated skeletal muscle morphology by H&E staining; quantified mRNA expression levels of Nrf2 and its downstream targets in the skeletal muscle by RT-qPCR; and determined protein abundance, localization, and expression patterns of Nrf2 and NOD-like receptor protein 3 (NLRP3) inflammasome by Western blotting and immunohistochemistry, respectively. In the skeletal muscle of IR mice, Nrf2 and its downstream targets were significantly down-regulated, whereas NLRP3 inflammasome was markedly up-regulated (*p* < 0.05 or *p* < 0.01). IR mice subjected to aerobic exercise exhibited reduced serum glucose and lipid levels together with a lower insulin-resistance index (*p* < 0.05 or *p* < 0.01); morphologically, inter-myofibrillar spaces were narrowed, intrafiber vacuoles diminished, and cellular integrity restored. Concomitantly, Nrf2 and its downstream targets were up-regulated, whereas NLRP3 inflammasome components were down-regulated in the skeletal muscle (*p* < 0.05 or *p* < 0.01). Intraperitoneal administration of luteolin during exercise, however, partially attenuated or reversed these exercise-induced improvements by inhibiting the activation of Nrf2 (*p* < 0.05 or *p* < 0.01). These results indicate that aerobic exercise confers protective effects against IR by activating the Nrf2 signaling pathway, thereby attenuating oxidative stress and inflammation; these benefits are markedly attenuated when Nrf2 activity is pharmacologically inhibited.

## 1. Introduction

Insulin resistance (IR) is a key pathophysiological mechanism underlying metabolic disorders. It is not only closely associated with the development and progression of type 2 diabetes mellitus (T2DM) [1] but also can induce a series of pathophysiological processes, such as dyslipidemia and endothelial dysfunction [2], thus posing a severe challenge to global public health. Oxidative stress and inflammatory responses play crucial roles in the development and progression of IR. Research has demonstrated that long-term consumption of a high-fat diet can lead to the sustained production of reactive oxygen species (ROS) and the release of a large number of pro-inflammatory factors in the body, thereby inducing oxidative stress and inflammatory responses. This process can damage several key components of the insulin signaling pathway, disrupt the normal physiological functions of insulin, and ultimately result in IR [3]. Garg et al. [4] and Sasaki et al. [5] reported that interventions with PSTi8 and running exercise in high-fat diet-fed mice reduced the secretion of ROS and downregulated the expression of pro-inflammatory factors, thereby inhibiting oxidative stress and inflammatory responses. These changes restored the Insulin Receptor Substrate-1 (IRS-1)/Phosphatidylinositol 3-Kinase (PI3K)/Protein Kinase B(Akt) signaling pathway and consequently ameliorated IR induced by the high-fat diet. These findings highlight the critical roles of oxidative stress and inflammation in IR, suggesting that inhibition of oxidative stress and inflammation may be an effective strategy for improving IR. Moreover, oxidative stress and inflammation are interconnected through a bidirectional positive feedback loop [6,7], which may amplify each other and thereby accelerate the pathological progression of IR.

Nuclear factor E2-related factor 2 (Nrf2) is a key transcription factor that is widely present in mammalian cells. Early studies have revealed that in mice models of IR induced by a high-fat diet, the Nrf2 signaling pathway is impaired. However, specific activation of the Nrf2 signaling pathway effectively ameliorated the state of IR [8]. Subsequently, the findings by Zhang et al. [9] and Pei et al. [10] revealed that activation of the Nrf2 signaling pathway through various approaches, including Lycium barbarum polysaccharides and Shenqi Tangluo pills, effectively lowers blood lipid and glucose levels in rats, leading to a significant improvement in IR. In the present study, luteolin (20 mg/kg) was used as a pharmacological inhibitor of the Nrf2 signaling pathway. Notably, as a natural flavonoid, luteolin exerts a bidirectional, dose- and cell-type-dependent regulatory effect on Nrf2, which is mainly characterized by activation at low concentrations and inhibition at high concentrations. Studies have shown that in C2C12 myocytes, luteolin can enhance the antioxidant defense capacity of myocytes by activating Nrf2, but the effective concentration window is narrow (6.25–12.5 µM). Excessively high concentrations may exert the opposite effect due to the pro-oxidative effects or cytotoxicity of flavonoids [11]. As early as 2011, Tang et al. first demonstrated via the ARE-luciferase reporter gene assay that luteolin could concentration-dependently inhibit Nrf2 activity in A549 human non-small cell lung cancer cells [12]. Subsequently, Yang et al. [13] and Tsai et al. [14] successively confirmed that luteolin could downregulate the expression of its downstream target gene Heme Oxygenase-1(HO-1) by inhibiting the Nrf2/ARE signaling pathway. The luteolin dosage adopted in this study was 20 mg/kg, which falls within the range of high-dose intervention. Consistent with the above pharmacological characteristics, this dosage exceeded the low-concentration activation window of Nrf2 in skeletal muscle and ultimately exerted an inhibitory effect on Nrf2 signaling in high-fat diet-induced insulin-resistant skeletal muscle.

Aerobic exercise is widely recognized as an economical and safe lifestyle intervention that can delay the onset and progression of IR through multiple pathways, primarily involving the activation of Nrf2, enhancement of cellular antioxidant capacity, and suppression of inflammatory responses [15]. Notably, the intervention effects of aerobic exercise are positively correlated with its duration [16,17]. Mechanistically, the beneficial effects of aerobic exercise on IR are closely associated with its regulation of the Nrf2-mediated antioxidant pathway and inflammatory signaling. Liu et al. reported that aerobic exercise increased nuclear Nrf2 levels and modulated iron homeostasis in hepatocytes of high-fat diet-induced hepatic IR mice, consequently upregulating insulin receptor and glucose transporter expression to enhance glucose uptake and storage and attenuate IR [18]. Further studies have supplemented that regular aerobic exercise can reinforce its improving effect on IR by increasing the protein expression of Nrf2 and its downstream antioxidant enzymes, enhancing cellular antioxidant capacity, and inhibiting the activity of pro-inflammatory cytokines [19,20,21,22]. However, previous studies have predominantly focused on elucidating the mechanisms by which aerobic exercise ameliorates IR through individual signaling pathways. Although numerous studies have confirmed that oxidative stress and inflammation can synergistically promote each other, and Nrf2 plays a key role in integrating and regulating these two pathological processes, the expression pattern of the Nrf2-oxidative stress-inflammation network in skeletal muscle, as well as the regulatory effect of aerobic exercise on this network—especially in the context of IR induced by a high-fat diet—has not been fully elucidated. To address this research gap, the present study employed a mouse model of IR induced by a high-fat diet, combined with aerobic exercise intervention and intraperitoneal injection of Luteolin, to systematically investigate the mechanism of action of the Nrf2-oxidative stress-inflammation network in exercise-induced prevention of IR, aiming to provide theoretical support and experimental evidence for the application of exercise intervention in high-fat diet-induced IR.

## 2. Result

### 2.1. Establish IR Model Mice by High-Fat Diet Feeding

**Figure 1:**
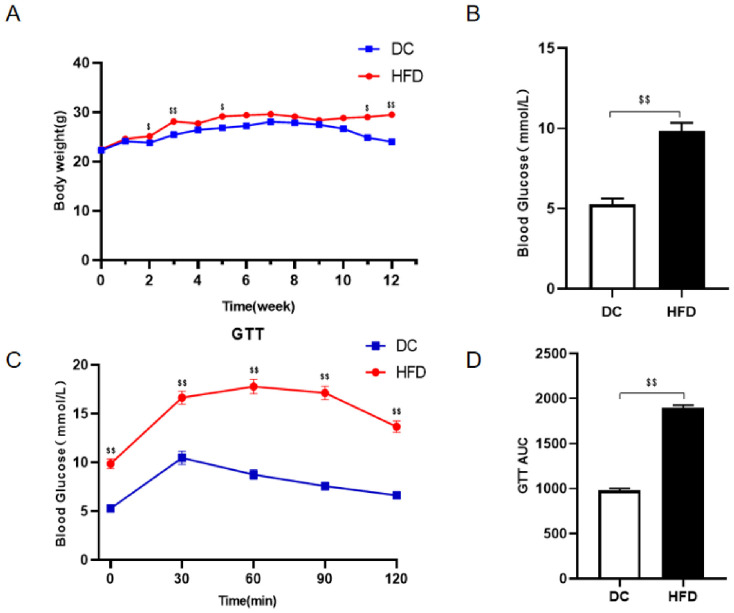
Establishment of Insulin resistance (IR) model mice. (**A**) represents the changes in body weight of mice during the modeling process; (**B**) represents the comparison of fasting blood glucose (FBG) levels in mice before and after modeling; (**C**) represents the comparison of glucose tolerance test (GTT) in mice before and after modeling; (**D**) represents the comparison of the area under the curve (AUC) of GTT in mice before and after modeling. ^$^ *p* < 0.05, ^$$^ *p* < 0.01 vs. DC.

After 12 weeks of high-fat feeding, body weight, fasting blood glucose, and glucose tolerance were measured in the mice. If there was a significant increase in body weight and fasting blood glucose (FBG), and glucose tolerance was impaired, the establishment of the IR model was considered successful [23]. As shown in Figure 1A,B, after 12 weeks of high-fat diet, the body weight and FBG levels of mice in the HFD group were markedly higher than those in the DC group (*p* < 0.01). The glucose tolerance test revealed that blood glucose levels of mice in the HFD group were significantly higher than those in the DC group at 0, 30, 60, 90, and 120 min (*p* < 0.01), indicating impaired glucose tolerance. Moreover, the area under the glucose tolerance curve (AUC) in the HFD group was notably higher than that in the DC group (*p* < 0.01), suggesting impaired glucose metabolism and reduced insulin sensitivity (As shown in Figure 1C,D). In summary, after 12 weeks of high-fat feeding, mice in the HFD group exhibited significant increases in body weight and fasting blood glucose levels, along with severely impaired glucose tolerance, indicating the successful establishment of the IR mouse model. Subsequently, the 32 mice that successfully underwent modeling were randomly divided into four groups: the IR model group (IR, *n* = 8), the exercise group (EX, *n* = 8), the Luteolin group (LUT, *n* = 8), and the exercise combined with Luteolin group (EL, *n* = 8). The mice in the IR group maintained their habitual lifestyle without any intervention. The EX group received 6 weeks of moderate-intensity treadmill exercise intervention (0°slope, 60 min/d, 5 d/w). The LUT group was subjected to intraperitoneal injection of luteolin at a dose of 20 mg/kg, 5 days per week. The EL group was simultaneously treated with treadmill exercise and intraperitoneal luteolin injection.

### 2.2. Aerobic Exercise Reduces the Blood Lipid, Blood Glucose Levels and HOMA-IR in IR Mice

**Figure 2:**
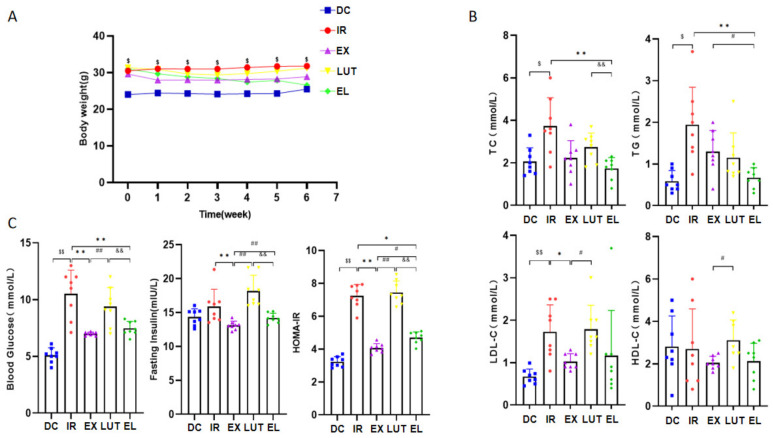
Changes in body weight, blood lipid, blood glucose and insulin resistance index of mice. (**A**) body weight changes in mice during the intervention period; (**B**) comparison of serum lipid levels in mice after intervention; (**C**) Comparison of FBG, insulin levels, and HOMA-IR in mice after intervention. *n* = 8 mice per group. ^$^ *p* < 0.05, ^$$^ *p* < 0.01 vs. DC; * *p* < 0.05, ** *p* < 0.01 vs. IR; ^#^ *p* < 0.05, ^##^ *p* < 0.01 vs. EX; ^&&^ *p* < 0.01 vs. LUT.

The experimental results showed (As shown in Figure 2B,C) that a long-term high-fat diet led to notably higher levels of total cholesterol (TC), triglycerides (TG), low-density lipoprotein cholesterol (LDL-C), FBG, and homeostasis model assessment of insulin resistance (HOMA-IR) in the IR group compared with the DC group (*p* < 0.05 or *p* < 0.01). After 6 weeks of aerobic exercise intervention, the levels of LDL-C, FBG, and HOMA-IR in the EX group were significantly lower than those in the IR group (*p* < 0.05 or *p* < 0.01). Moreover, during the aerobic exercise intervention, intraperitoneal injection of Luteolin resulted in a reduced significantly HOMA-IR in the EL group compared with the IR group (*p* < 0.05), but it was still markedly higher than that in the EX group (*p* < 0.05).

### 2.3. Aerobic Exercise Improves the Muscle Tissue Morphology of IR Mice

**Figure 3:**
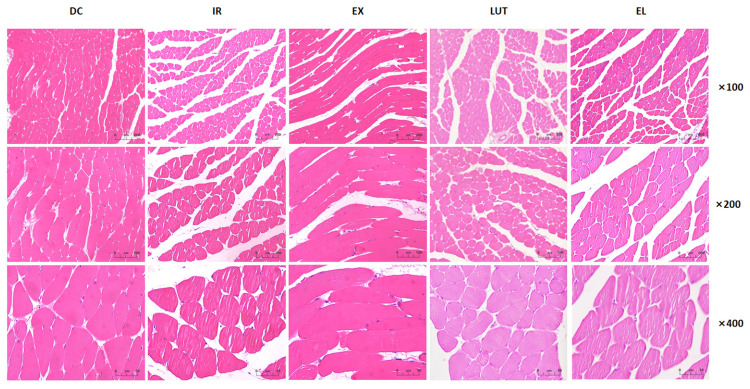
H&E staining of skeletal muscle. The scale bars were 200 μm, 100 μm and 50 μm for 100×, 200× and 400× magnifications, respectively.

**Figure 4:**
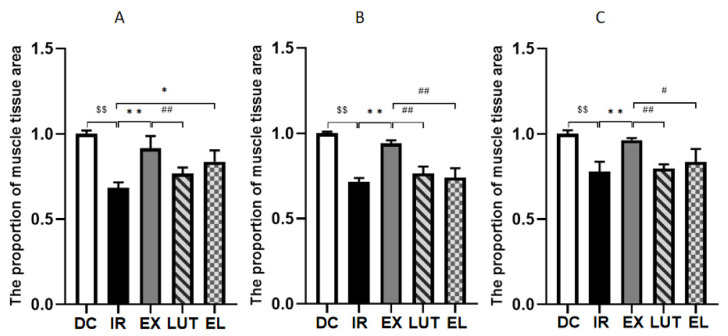
The proportion of muscle tissue area. (**A**–**C**) represent the proportions of the muscle tissue area at 100×, 200×, and 400× magnifications, respectively. ^$$^ *p* < 0.01 vs. DC; * *p* < 0.05, ** *p* < 0.01 vs. IR; ^#^ *p* < 0.05, ^##^ *p* < 0.01 vs. EX.

The results of H&E staining showed (As shown in Figure 3 and Figure 4) that the muscle tissue structure in the DC group was clear, with neatly arranged muscle fibers and fewer internal cavities. Compared with the DC group, the IR group showed widened intermyofibrillar spaces and increased intracellular cavities, accompanied by a significant reduction in the proportional area of muscle tissue (*p* < 0.01). Compared with the IR group, the EX group exhibited decreased intermyofibrillar spaces and fewer internal cavities, along with better-preserved cellular ultrastructure and a significant increase in the proportional area of muscle tissue (*p* < 0.01). In contrast, the LUT group displayed loosely arranged myocytes, irregular tissue morphology, and an increased number of intracellular cavities within the myofibers, with the proportional area of muscle tissue being significantly lower than that in the EX group (*p* < 0.01). The EL group presented better-aligned myofibers but still retained a relatively large number of intracellular cavities. Meanwhile, in the fields of view at 100× magnification, the proportional area of muscle tissue in the EL group was markedly higher than that in the IR group (*p* < 0.05). At 200× and 400× magnifications, its proportional area of muscle tissue was significantly reduced compared with the EX group (*p* < 0.05 or *p* < 0.01). These findings indicate that a long-term high-fat diet induces pathological alterations in muscle tissue, and 6 weeks of aerobic exercise can alleviate such histopathological damage.

### 2.4. Aerobic Exercise Modulates the Expression of Nrf2 and Its Downstream Genes

**Figure 5:**
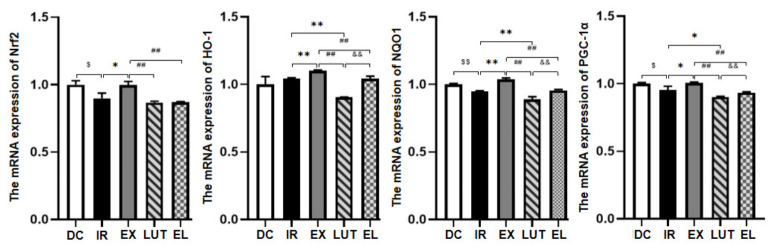
Real-time qPCR analysis was performed to detect the mRNA levels of Nrf2 and its related factors in the skeletal muscle. *n* = 8 mice per group. ^$^ *p* < 0.05, ^$$^ *p* < 0.01 vs. DC; * *p* < 0.05, ** *p* < 0.01 vs. IR; ^##^ *p* < 0.01 vs. EX; ^&&^ *p* < 0.01 vs. LUT.

**Figure 6:**
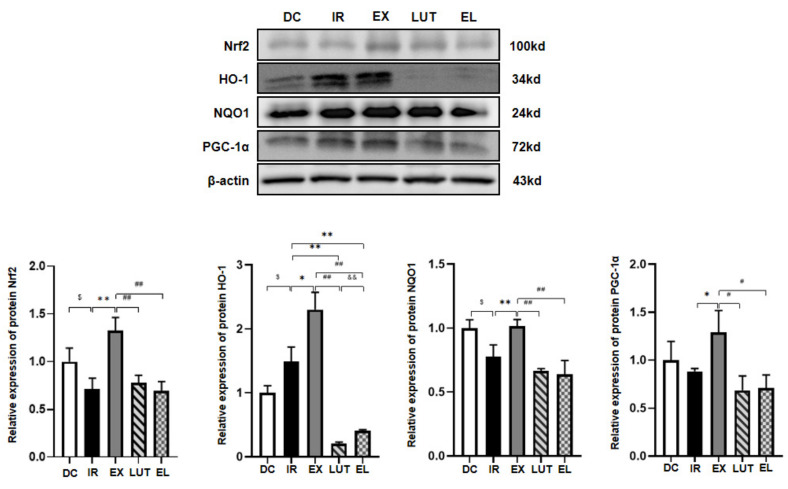
Western blotting analysis was performed to detect the protein expression levels of Nrf2 and its related factors in the skeletal muscle. *n* = 8 mice per group. ^$^ *p* < 0.05 vs. DC; * *p* < 0.05, ** *p* < 0.01 vs. IR; ^#^ *p* < 0.05, ^##^ *p* < 0.01 vs. EX; ^&&^ *p* < 0.01 vs. LUT.

As shown in Figure 5 and Figure 6, quantitative real-time PCR (qPCR) and Western blotting analyses revealed that, compared with the DC group, the IR group exhibited significantly reduced mRNA and protein levels of Nrf2 and NAD(P)H: Quinone Oxidoreductase 1 (NQO1) (*p* < 0.05 or *p* < 0.01). The mRNA expression of Peroxisome proliferator-activated receptor gamma coactivator 1-alpha (PGC-1α) was also markedly downregulated (*p* < 0.05), while no significant change was observed in its protein abundance (*p* > 0.05). Notably, the protein expression of HO-1 was significantly upregulated in the IR group(*p* < 0.05). Compared with the IR group, six weeks of aerobic exercise significantly upregulated both the mRNA and protein levels of Nrf2, HO-1, NQO1, and PGC-1α in the EX group (*p* < 0.05 or *p* < 0.01). These data indicate that aerobic exercise effectively activates the Nrf2 signaling pathway at both transcriptional and post-translational levels in skeletal muscle under IR conditions. Luteolin alone exerted no significant regulatory effect on both mRNA and protein expression of Nrf2 itself (*p* > 0.05). However, it markedly inhibited the transcriptional levels of Nrf2 downstream target genes, including HO-1, NQO1 and PGC-1α, and significantly downregulated the protein expression of HO-1 (*p* < 0.05 or *p* < 0.01). Compared with the EX group, the mRNA and protein levels of Nrf2, HO-1, NQO1, and PGC-1α were significantly decreased in the EL group (aerobic exercise combined with luteolin intervention) (*p* < 0.05 or *p* < 0.01). Collectively, these results suggest that under IR conditions, luteolin intervention weakens the beneficial regulatory effects of aerobic exercise on the mRNA and protein expression of Nrf2, HO-1, NQO1, and PGC-1α.

### 2.5. Aerobic Exercise Regulates the Expression of NLRP3 and Related Inflammatory Factors

**Figure 7:**
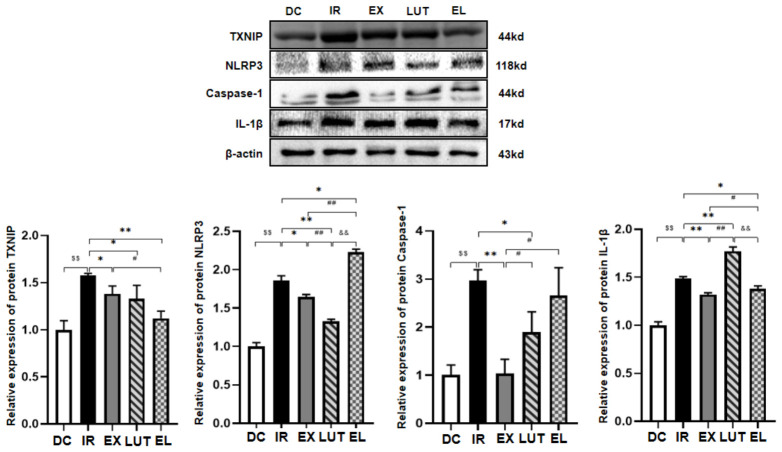
Western blotting analysis was performed to detect the protein expression levels of NLRP3 and its related factors in the skeletal muscle. *n* = 8 mice per group. ^$$^ *p* < 0.01 vs. DC; * *p* < 0.05, ** *p* < 0.01 vs. IR; ^#^ *p* < 0.05, ^##^ *p* < 0.01 vs. EX; ^&&^ *p* < 0.01 vs. LUT.

As shown in Figure 7, Western blotting analysis of inflammatory factors demonstrated that the relative protein expression levels of thioredoxin-interacting protein (TXNIP), NLRP3, cysteine-aspartic acid protease 1(Caspase-1), and Interleukin-1 beta (IL-1β) in the IR group were significantly higher than those in the DC group (*p* < 0.01). Compared with the IR group, the 6-week aerobic exercise intervention significantly reduced the relative protein expression levels of TXNIP, NLRP3, caspase-1, and IL-1β in mice of the EX group (*p* < 0.05 or *p* < 0.01). Combined intervention with exercise and luteolin resulted in significantly lower protein expression levels of TXNIP and IL-1β in the EL group compared with the IR group (*p* < 0.05 or *p* < 0.01). However, the relative protein expression levels of NLRP3, caspase-1, and IL-1β in the EL group were significantly higher than those in the EX group (*p* < 0.05 or *p* < 0.01).

**Figure 8:**
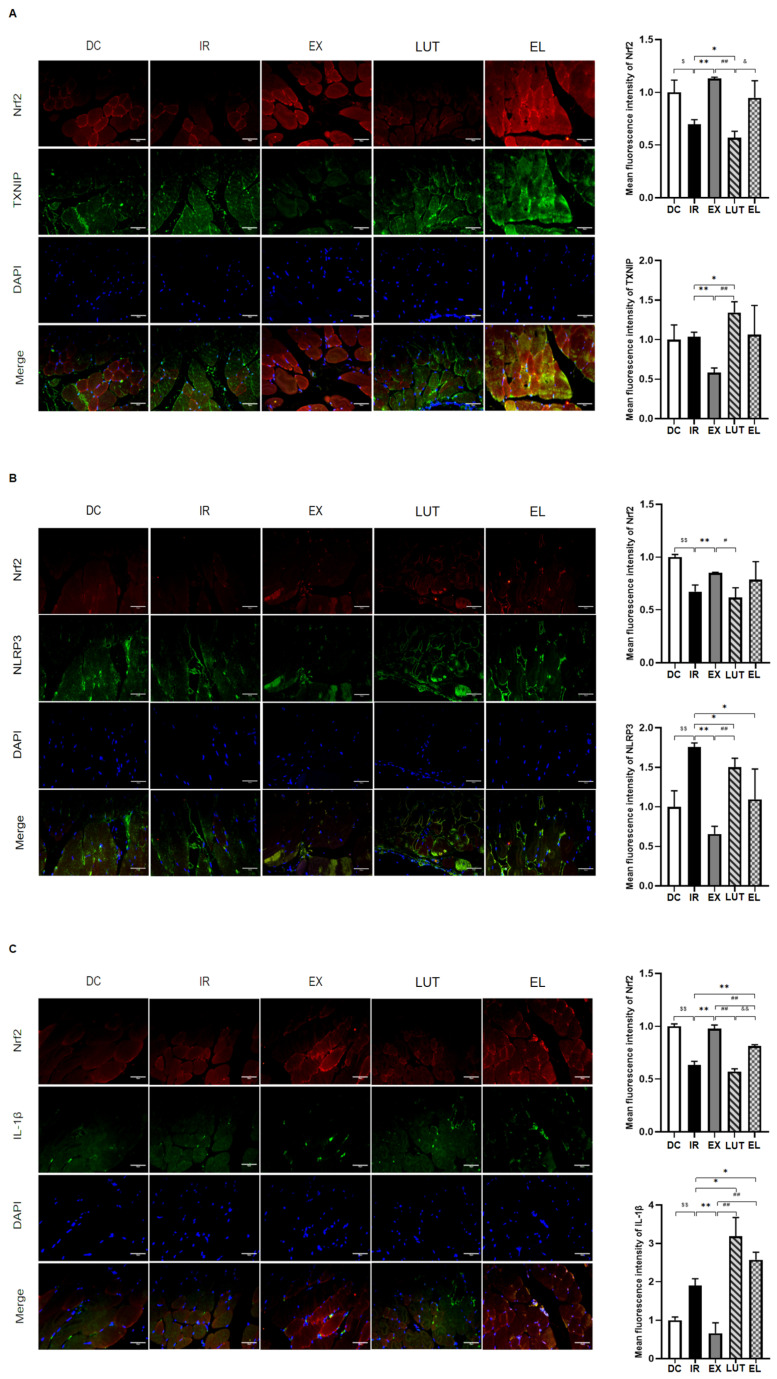

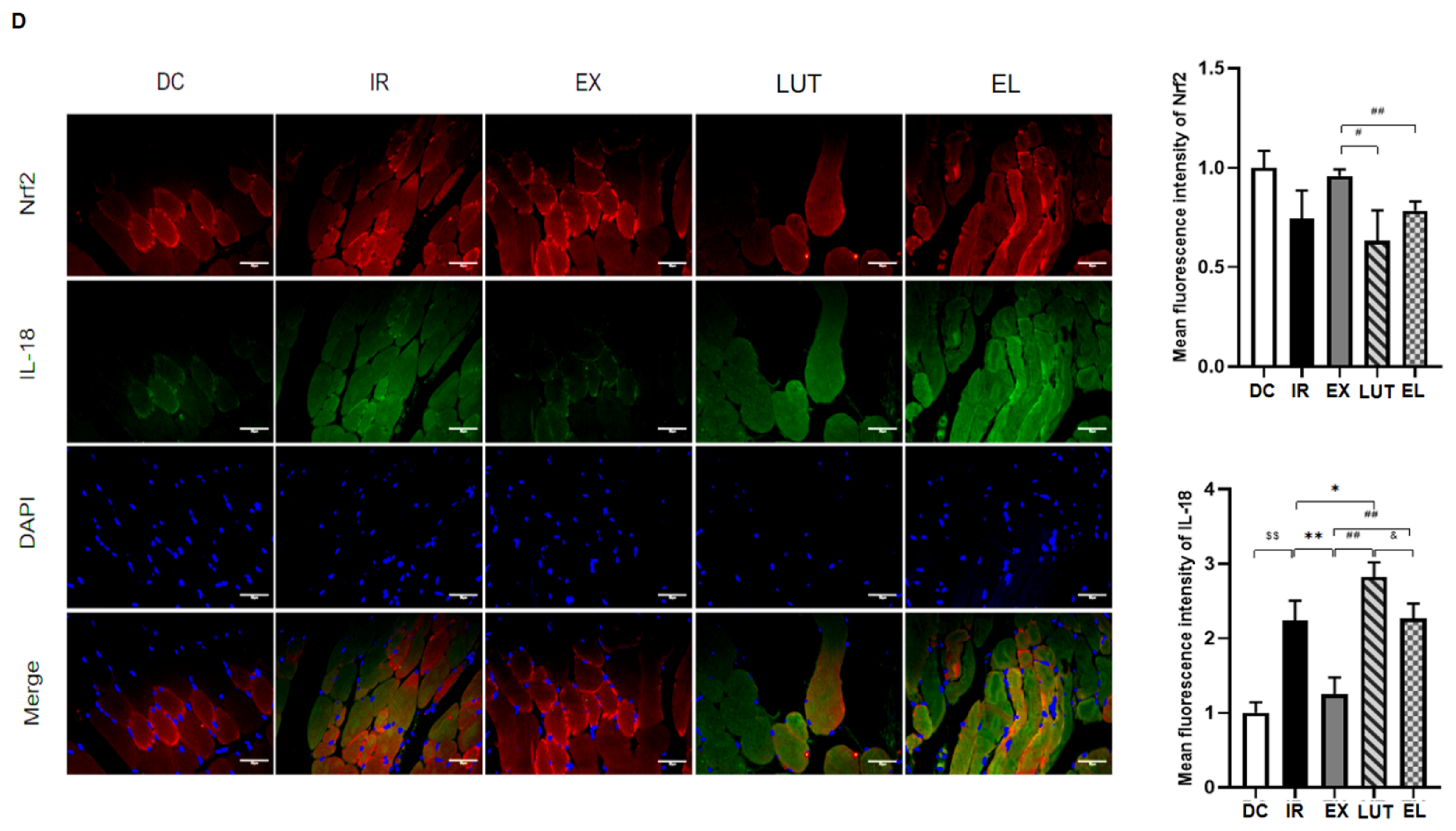
The results of immunofluorescence staining. (**A**) 400× immunofluorescence staining for Nrf2 (red) and TXNIP (green). (**B**) 400× immunofluorescence staining for Nrf2 (red) and NLRP3 (green). (**C**) 400× immunofluorescence staining for Nrf2 (red) and IL-1β (green). (**D**) 400× immunofluorescence staining for Nrf2 (red) and IL-18 (green). Scale bar = 50 μm. *n* = 8 mice per group.^$^ *p* < 0.05, ^$$^ *p* < 0.01 vs. DC; * *p* < 0.05, ** *p* < 0.01 vs. IR; ^#^ *p* < 0.05, ^##^ *p* < 0.01 vs. EX; ^&^ *p* < 0.05, ^&&^ *p* < 0.01 vs. LUT.

As shown in Figure 8, immunofluorescence staining analysis revealed that, compared with the DC group, the IR group exhibited significantly increased mean fluorescence intensity (MFI) of NLRP3, IL-1β, and Interleukin-18 (IL-18) (*p* < 0.01). Conversely, following 6 weeks of aerobic exercise intervention, the EX group exhibited a significant reduction in the MFI of TXNIP, NLRP3, IL-1β, and IL-18 (*p* < 0.01). In addition, concurrent use of Luteolin to inhibit Nrf2 activity during exercise intervention significantly attenuated the inhibitory effects of exercise on NLRP3 and its downstream inflammatory factors. The aforementioned results indicate that the overactivation of chronic inflammation is closely associated with disease progression. 6 weeks of aerobic exercise can exert a protective effect against disease by suppressing the inflammatory response.

### 2.6. The Association Between Nrf2 Activity and the Inflammatory Response

**Figure 9:**
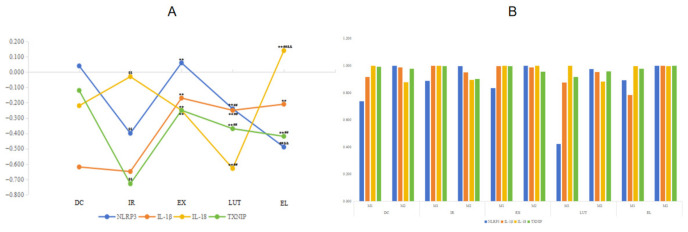
Spatial co-localization relationship between Nrf2 and inflammatory indicators. A represents the Pearson correlation coefficient between Nrf2 and the inflammatory markers. (**A**) Pearson’s R value closer to 1 indicates a stronger positive spatial correlation between the two proteins within the cell, meaning that regions with strong Nrf2 signaling also exhibit strong inflammatory marker signaling. Conversely, a Pearson’s R value closer to -1 indicates a stronger negative spatial correlation, meaning that regions with strong Nrf2 signaling exhibit weak inflammatory marker signaling. ^$$^ *p* < 0.01 vs. DC; ** *p* < 0.01 vs. IR; ^##^ *p* < 0.01 vs. EX; ^&&^ *p* < 0.01 vs. LUT. (**B**) represents the Manders’ colocalization coefficients between Nrf2 and the inflammatory markers. M1 coefficient indicates the proportion of the Nrf2 (red) signal that overlaps with the inflammatory marker (green) signal (i.e., the overlap ratio of Nrf2 to the inflammatory marker). M2 coefficient indicates the proportion of the inflammatory marker (green) signal that overlaps with the Nrf2 (red) signal (i.e., the overlap ratio of the inflammatory marker to Nrf2). Colocalization was considered present when the M1/M2 coefficient was >0.6, and absent when <0.3.

Pearson correlation analysis (Figure 9A) demonstrated that compared with the DC group, the IR group exhibited significantly stronger negative spatial correlations between Nrf2 and NLRP3, IL-1β, and TXNIP. Notably, aerobic exercise intervention markedly attenuated these negative correlation relationships. It is important to emphasize that spatial negative correlatioScale bar = 50 μmn cannot be directly equated with functional regulatory relationships; however, this distinctive distribution pattern suggests a potential association between altered Nrf2 activity and the activation of inflammatory signaling pathways. Manders’ colocalization analysis further revealed (Figure 9B) that Nrf2 and the inflammatory factors exhibited a high degree of colocalization across all experimental groups (M1/M2 > 0.6). It is noteworthy that upon treatment with the Nrf2 inhibitor (luteolin), the spatial co-localization ratio between Nrf2 and NLRP3 was significantly reduced, suggesting that Nrf2 activity may influence its intracellular distribution characteristics, in addition to its potential effects on expression level. Although the spatial analysis results are insufficient to establish a definitive causal relationship, these findings provide valuable insights for further investigation into the spatial mechanisms underlying the potential association between Nrf2 and inflammatory regulation.

## 3. Discussion

IR can disrupt normal metabolic processes, affecting multiple systems and organs, and significantly increasing the risk of chronic disease development [24]. High-fat diet alters adipose tissue distribution, perturbs lipid metabolism, and reduces insulin sensitivity, representing one of the critical factors exacerbating IR [25]. Li et al. demonstrated that a 12-week high-fat diet regimen resulted in marked weight gain, glucose intolerance, and reduced insulin sensitivity, leading to significant exacerbation of IR in mice. Conversely, 8-week treadmill exercise training substantially ameliorated body weight and glucose intolerance, enhanced insulin sensitivity, and effectively attenuated IR progression [16]. Our findings corroborate the observations reported by Li et al., demonstrating that chronic high-fat diet feeding significantly elevated serum TG, TC, and LDL-C levels, as well as fasting blood glucose concentrations, concomitant with increased HOMA-IR values in mice. Importantly, 6-week aerobic exercise training effectively reversed these metabolic aberrations, attenuating the onset and progression of IR. Skeletal muscle plays a pivotal role in IR; it constitutes not only the primary site where IR first manifests, but also the preferred therapeutic target for restoring whole-body glucose homeostasis [26]. Prolonged high-fat diet exposure elicits systemic hyperglycemia and promotes ectopic lipid deposition within the skeletal muscle. This pathological cascade not only compromises skeletal muscle metabolic homeostasis but also accelerates the onset and progression of IR [27]. Previous studies have demonstrated that 8-week treadmill training mitigates skeletal-muscle architectural disarray associated with type 2 diabetes and robustly ameliorates glucolipid metabolic dysfunction, leading to a marked attenuation of IR [28]. In the present study, skeletal muscle fibers of IR mice exhibited marked disarray and increased intramuscular cavitation. 6 weeks of aerobic exercise restored fiber alignment and reduced cavitation, corroborating the therapeutic efficacy of exercise in ameliorating IR-induced myopathology.

Nrf2 represents a pivotal transcription factor that orchestrates the cellular antioxidant defense machinery [29]. Upon activation, Nrf2 markedly upregulates the protein expression of its downstream target genes, including HO-1 and NQO1, thereby collaboratively maintaining intracellular redox homeostasis and preserving normal cellular physiological functions [30]. HO-1 catalyzes the enzymatic cleavage of heme to generate carbon monoxide, biliverdin, and free iron, thereby exerting robust antioxidant, anti-inflammatory, and cytoprotective effects [31]. NQO1, a pivotal antioxidant flavoprotein, attenuates intracellular oxidative stress by catalyzing the two-electron redox turnover of NAD(P)H, thereby contributing to the amelioration of insulin sensitivity [32]. Accumulating evidence demonstrates that up-regulation of the Nrf2/HO-1/NQO1 axis attenuates oxidative-stress-induced damage, potentiates insulin sensitivity, and consequently ameliorates IR [10,33]. Under normal physiological conditions, Nrf2 binds to Keap1 and forms a stable complex that is sequestered in the cytoplasm. As a critical cytoplasmic repressor of Nrf2, Keap1 assembles with Cullin-3 and RING-box protein 1 to constitute a functional E3 ubiquitin ligase complex. This machinery ultimately mediates the ubiquitination and proteasomal degradation of Nrf2, thereby maintaining Nrf2 at a low basal expression level under steady-state conditions [34,35]. As a crucial physiological stimulus, exercise elevates the intracellular AMP/ATP ratio and subsequently activates AMPK, a core cellular energy sensor. Activated AMPK directly phosphorylates Nrf2, thereby promoting its nuclear translocation and enhancing transcriptional activity. This signaling cascade ultimately triggers the endogenous antioxidant defense response [36]. Related studies have found that 12 weeks of swimming exercise can activate Nrf2 through the SIRT1/AMPK cascade, initiate the transcription program of antioxidant genes, and inhibit oxidative stress [37]. Subsequent studies further revealed that in the IR mouse model induced by a high-fat diet, regular aerobic exercise could increase the nuclear Nrf2 content, upregulate the expression of iron transporter-1, thereby promoting iron homeostasis, and ultimately enhancing insulin sensitivity and slowing down the progression of insulin resistance induced by a high-fat diet [18]. The present study provides novel evidence that aerobic exercise counteracts high-fat-diet-induced IR. Specifically, 6 weeks of aerobic training up-regulated the skeletal-muscle Nrf2/HO-1/NQO1 axis, reinforced systemic antioxidant capacity, and reversed the IR phenotype imposed by the high-fat regimen.

It is worth noting that the role of HO-1 in IR is complex, as it not only has potential mechanisms to improve IR but also exerts complex effects that promote IR. Early studies have shown that in the IR rat model established by high-sugar and high-fat diet feeding, the mRNA and protein expression levels of Nrf2 and HO-1 are significantly decreased [38]. Subsequently, Pei et al. observed in a T2DM mouse model that the protein expression levels of Nrf2 and its downstream target factors HO-1 and NQO1 in skeletal muscle were significantly reduced [10]. Unlike the above-mentioned research, as early as 2014, Jais et al. observed a significant increase in the expression of HO-1 in the liver and adipose tissue of obese patients with IR, and the expression level was directly correlated with the severity of metabolic disorders. Further studies combined clinical data with genetic evidence from five tissue-specific HO-1 knockout models, overexpression models, and gene repair scenarios, and found that HO-1 exerts a significant pro-inflammatory effect in metabolic control, thereby interfering with the insulin signaling pathway and driving the development of IR in mice or humans [39]. Furthermore, the results of the present study demonstrated that in the mouse IR model induced by a HFD, the expression of Nrf2 was significantly downregulated, whereas the protein expression level of HO-1 was markedly upregulated. The potential mechanism underlying this phenomenon may be as follows: in addition to the canonical Nrf2/ARE signaling pathway, the HO-1 gene promoter contains binding sites for various transcription factors, such as activator protein-1 (AP-1) and hypoxia-inducible factor (HIF), which can be independently activated under different stress conditions [40]. Studies by Yeligar et al. have confirmed that ethanol-induced HO-1 expression is coordinately regulated by three signaling pathways, namely the Nrf2/ARE, HIF-1α/hypoxia response element (HRE), and AP-1 pathways, whereas NQO1 is mediated solely by the Nrf2/ARE pathway [41]. This differential regulatory pattern indicates that the HO-1 promoter possesses the characteristic of responding to multiple transcription factors and can be independently activated by HIF-1α or AP-1 in the absence of Nrf2, suggesting the existence of an Nrf2-independent regulatory pathway for HO-1.

Under normal physiological conditions, TXNIP forms a stable complex with thioredoxin (TRX), thereby preserving intracellular redox homeostasis. Upon oxidative stress, TXNIP dissociates from TRX and rapidly associates with the NLRP3 inflammasome, initiating its activation cascade [42,43]. The NLRP3 inflammasome is a multiprotein complex that serves as a critical intracellular sensor of both endogenous and exogenous danger signals. Upon activation, it facilitates the autocatalytic cleavage of pro-caspase-1 into its active form, which subsequently mediates the proteolytic maturation and secretion of the proinflammatory cytokines IL-1β and IL-18, thereby orchestrating a robust inflammatory response [44]. IL-1β and IL-18 propagate inflammatory cascades through the activation of distinct proinflammatory signaling pathways, which subsequently attenuate IRS phosphorylation and disrupt insulin signal transduction, ultimately culminating in the development of IR [45]. Studies have demonstrated that TXNIP, NLRP3 inflammasome, and their downstream pro-inflammatory cytokines are significantly upregulated in polycystic ovary syndrome rats with IR. Inhibition of the TXNIP/NLRP3 signaling cascade effectively attenuates chronic inflammation and concomitantly ameliorates IR [46]. Studies by Bian [47] and Ji [48] have demonstrated that the NLRP3 inflammasome and its downstream pro-inflammatory cytokines IL-1β and IL-18 are significantly upregulated in the hippocampus of IR mice. 12 weeks of progressive treadmill training and resistance exercise effectively suppressed NLRP3 inflammasome activation and attenuated IL-1β and IL-18 expression, thereby ameliorating hippocampal neuroinflammation in IR mice. Our findings provide further evidence that high-fat diet-induced IR significantly upregulates the expression of key components of the NLRP3 inflammasome pathway, including TXNIP, NLRP3, cleaved caspase-1, and IL-1β in the skeletal muscle, indicating enhanced NLRP3 inflammasome activation. Six weeks of aerobic exercise training effectively suppressed TXNIP expression, concomitantly reduced NLRP3 inflammasome component levels, and inhibited caspase-1 activation and subsequent IL-1β production, thereby attenuating inflammation and improving IR.

Previous studies have shown that an 8-week aerobic exercise intervention significantly alleviates vascular inflammation in rats with type 2 diabetes. The underlying molecular mechanisms may involve the activation of the Nrf2 signaling pathway, which in turn mitigates oxidative stress [49]. Lei et al. further elucidated that 8 weeks of aerobic exercise training effectively improves glucose homeostasis in type 2 diabetic rat models, likely through the Nrf2/HO-1 signaling cascade-mediated restoration of skeletal muscle redox balance [50]. Collectively, these findings suggest that Nrf2-mediated regulation of oxidative stress and inflammatory responses may play a pivotal role in exercise-induced protection against metabolic disorders. Our present data demonstrate that 6 weeks of aerobic exercise effectively activates the Nrf2 signaling pathway, reverses the high-fat diet-induced decline in antioxidant enzyme activities, including NQO1, and suppresses the aberrant overexpression of the NLRP3 inflammasome and its downstream effectors IL-1β and IL-18. These molecular adaptations ultimately ameliorate glucose and lipid metabolic dysfunction and pathological alterations in the skeletal muscle, consequently attenuating the progression of IR. To elucidate the role of Nrf2 in exercise-mediated attenuation of oxidative stress and inflammatory responses, as well as its contribution to the improvement of metabolic disorders such as IR, Merry et al. conducted a study involving 6 weeks of treadmill exercise training in both Nrf2-deficient mice and wild-type controls. Their results revealed that, compared to wild-type mice, Nrf2-deficient mice exhibited significantly elevated levels of TG, hyperglycemia, and increased free fatty acids. Additionally, Nrf2-deficient mice displayed impaired glucose homeostasis and heightened hepatic oxidative stress markers, highlighting the critical role of Nrf2 in mediating the beneficial metabolic effects of exercise [51]. These findings indicate that Nrf2 deletion may compromise the therapeutic efficacy of exercise against metabolic disorders. In contrast, this study adopted a pharmacological approach via intraperitoneal injection of luteolin to regulate exercise-induced Nrf2 signaling transduction. Our results demonstrated that pharmacological intervention with luteolin during aerobic exercise markedly blunted exercise-mediated Nrf2 signaling activation in IR model mice. This intervention reduced the protein levels of Nrf2 downstream targets HO-1 and NQO1, accompanied by intensified NLRP3 inflammasome activation and elevated expression of pro-inflammatory cytokines IL-1β and IL-18 in skeletal muscle. The transient inhibitory effect of luteolin on Nrf2 observed in our study is restricted to the combined intervention context of high-fat diet stress and aerobic exercise co-treatment, rather than a universal inhibitory property of luteolin. Collectively, such context-related Nrf2 signaling restriction partially abrogated the protective effects of aerobic exercise, including the alleviation of oxidative stress and inflammation, and ultimately weakened exercise-mediated improvements in glucolipid metabolism disorder, skeletal muscle pathological damage, and IR deterioration. It should be noted that, due to the pleiotropic effects of luteolin and the absence of direct assessment of Nrf2 activity in the present study, we cannot definitively establish a causal relationship between Nrf2 inhibition and the observed alterations in inflammatory responses. Nevertheless, when combined with the findings reported by Merry et al., our results collectively indicate that Nrf2 may serve as a crucial mediator underlying the exercise-induced protective effects against metabolic disorders. Therefore, further studies incorporating direct assessment of Nrf2 activity are warranted to verify this potential regulatory role of Nrf2.

**Figure 10:**
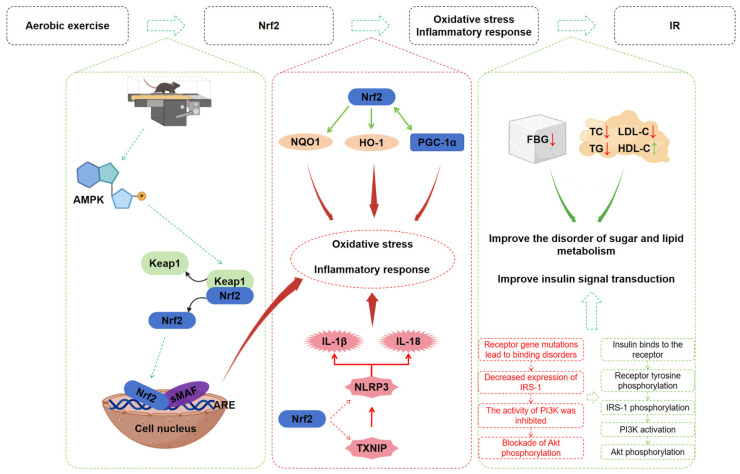
Proposed hypothetical schematic model illustrating the associative relationships among aerobic exercise, Nrf2 signaling, oxidative stress/inflammatory responses, and IR in the present study. Solid arrows: Associations validated experimentally in this study. Dashed arrows: Hypothesized regulatory pathways supported by published literature but not directly validated herein. Green arrows: Positive associations. Red arrows: Negative associations.

As illustrated in Figure 10, our results demonstrate that aerobic exercise training enhances Nrf2 signaling in skeletal muscle, which subsequently upregulates the expression of downstream antioxidant proteins HO-1 and NQO1. Meanwhile, aerobic exercise suppresses the expression of NLRP3 inflammasome components, cleaved caspase-1 and IL-1β, thereby alleviating oxidative stress and inflammatory responses and delaying the progression of IR.

## 4. Materials and Methods

### 4.1. General Protocol

**Figure 11:**
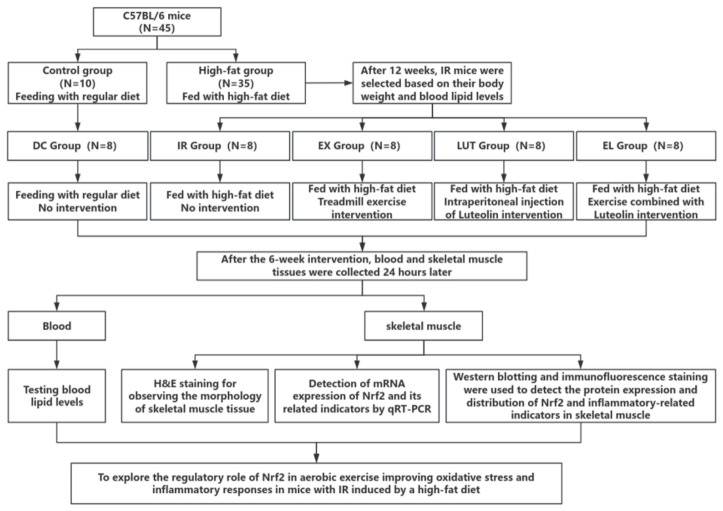
Flow chart.

The present study aims to investigate the regulatory role of Nrf2 in ameliorating oxidative stress and inflammatory responses in high-fat diet-induced insulin-resistant mice through aerobic exercise. The detailed information can be found in Figure 11.

### 4.2. Experimental Animals

The experiment utilized 45 male C57BL/6 mice at 7 weeks of age, with a body weight of 20–22 g. The mice were purchased from the Experimental Animal Center of Three Gorges University (License No.: SCXK (E) 2022-0012) and were housed at the Animal Center of Yangtze University. Housing conditions: The mice were maintained at a room temperature of 22 ± 2 °C, with a relative humidity of 50–60%, under a natural light-dark cycle, and provided with free access to food and water. This study was conducted in accordance with the “Regulations for the Administration of Affairs Concerning Experimental Animals” and was approved by the Ethics Committee of the Faculty of Medicine, Yangtze University (Approval No.: 202401025, Date: 10 July 2024).

### 4.3. Establishment of IR Model Mice

According to the experimental design, all mice were acclimated to the laboratory environment for 1 week and then randomly assigned into two groups: the control group (DC, *n* = 10) and the high-fat diet group (HFD, *n* = 35). The standard diet and high-fat diet (12492M, 60% fat content, purified type, product code: Boaigang-12492M) were both purchased from Beijing Boai Gang Biotechnology Co., Ltd. (Beijing, China) Mice in the HFD group were used to establish an IR model via a high-fat diet, and their body weight was measured weekly. After 12 weeks, mice were fasted for 16 h and then subjected to a glucose tolerance test (GTT) to assess the establishment of IR modeling. Ultimately, the 32 mice that successfully underwent modeling were randomly divided into four groups: the IR model group (IR, *n* = 8), the exercise group (EX, *n* = 8), the Luteolin group (LUT, *n* = 8), and the exercise combined with Luteolin group (EL, *n* = 8).

### 4.4. Intervention Plan

The exercise intervention [52] and the intraperitoneal injection protocol of Luteolin [13] were both modified based on the relevant references. During the intervention period, mice in the EX, LUT, and EL groups were maintained on a high-fat diet. Throughout the experimental period, body weight changes and the general health status of the mice in each group were closely monitored.

The EX group underwent a 6-week moderate-intensity treadmill exercise intervention. Initially, mice in the EX group performed an adaptive treadmill exercise. The exercise intensity was 10 m/min for the first 3 days and 12 m/min for the following 2 days, with each session lasting 30 min per day for a total of 5 days. Following the completion of the adaptive exercise, the mice commenced a 6-week regimen of moderate-intensity treadmill exercise, characterized by a gradient of 0, an exercise intensity of 12 m/min, a duration of 60 min per day, and a frequency of 5 days per week. The exercise sessions were scheduled between 5:00 PM and 7:00 PM. Mice in the LUT group were administered Luteolin (HY-N0162, MCE, Shanghai, China) via intraperitoneal injection. The dosage of the inhibitor was adjusted according to the body weight of the mice at 20 mg/kg. The injection schedule was synchronized with the exercise regimen of the EX group, lasting for 6 weeks with 5 injections per week. Mice in the EL group underwent both treadmill exercise and inhibitor injection interventions. The specific parameters of the treadmill exercise and intraperitoneal injection of Luteolin were consistent with those of the EX and LUT groups, respectively.

### 4.5. Organizing the Collection and Processing of Materials

24 h after the final intervention, mice in all groups were fasted for 12 h with free access to water. Before tissue collection, the mice were weighed, and whole blood was collected via orbital bleeding. After resting at room temperature for 1 h, the whole blood was centrifuged at 3000 rpm for 20 min to collect the supernatant for subsequent use. Simultaneously, skeletal muscle tissues were rapidly harvested from the mice. A portion of the tissues was placed in fixative (for morphological analysis), while the remaining tissues were immediately plunged into liquid nitrogen for rapid freezing and subsequently transferred to a −80 °C freezer for subsequent molecular biological assays.

#### 4.5.1. Blood Lipid and Blood Sugar Tests, Glucose Tolerance Test, and Calculation of Insulin Resistance Index

TC, TG, LDL-C, and high-density lipoprotein cholesterol (HDL-C) were determined using commercially available assay kits (Beyotime Biotechnology, Shanghai, China) according to the manufacturer’s instructions.

FBG and GTT: on the evening prior to the test, the mice were fasted with free access to water. Fresh bedding was also provided to minimize potential environmental influences on the test results. The following morning at 8:00 AM, FBG was measured. The glucometer was calibrated, and a new test strip was inserted to ensure accurate measurement. The tail of the mouse was gently restrained, and blood was collected from the tail vein. The blood was then applied to a glucometer test strip. The blood glucose level was recorded after the glucometer displayed a stable reading. After a 30 min acclimation period, the mice were gavaged with glucose solution (dose: 1 g/kg, concentration: 0.1 g/mL). Blood glucose levels were measured and recorded at 30, 60, 90, and 120 min post-gavage. After the procedure, the site of tail vein blood collection in the mice was disinfected before returning the mice to their cages, where food and water were provided to ensure proper recovery and nutrition.

The formula for calculating the HOMA-IR index is as follows: fasting insulin (μU/mL) × fasting blood glucose (mmol/L)/22.5.

#### 4.5.2. Hematoxylin and Eosin (H&E) Staining

Skeletal muscle tissues at identical anatomical sites were dissected from mice, adequately fixed, and trimmed into tissue blocks of uniform size. Residual fixative was removed by rinsing with pre-cooled PBS buffer. Tissues were subjected to gradient ethanol dehydration, followed by twice xylene transparency and paraffin embedding. Serial sections were prepared at 4 μm thickness. Sections were baked at 56–58 °C for 60 min, fully dewaxed in xylene, and rinsed with absolute ethanol and PBS. Hematoxylin nuclear staining was performed for 3–5 min; differentiation was conducted with hydrochloric acid alcohol under microscopic observation, followed by sufficient bluing in weak alkaline water. After gradient ethanol dehydration, eosin counterstaining was performed, and sections were cleared with xylene. Finally, sections were mounted with neutral gum after complete drying. For histological evaluation, more than five non-overlapping random high-magnification visual fields were captured per section under an optical microscope at unified magnification, with biological replicates set to ensure the reliability of pathological analysis.

#### 4.5.3. Immunofluorescence Staining Analysis

The sections were dewaxed in xylene I and II (15 min each), rehydrated in gradient ethanol (anhydrous I, anhydrous II, 95%, 85%, 75%; 5 min each), and rinsed with sterile PBS (pH 7.2–7.4). Antigen retrieval was performed with citrate buffer (pH 6.0) or EDTA buffer (pH 8.0) in a pressure cooker, followed by natural cooling to room temperature. After PBS washing, sections were blocked with 10% normal goat serum at room temperature for 60 min. Primary antibody (1:1000) was incubated overnight at 4 °C in a humidified dark chamber. The next day, after PBS rinsing, fluorescent-conjugated secondary antibody (1:200–1:500) was incubated at 37 °C for 1 h in the dark. Nuclei were stained with DAPI (room temperature, 5–10 min); excess DAPI was washed with PBS, and sections were mounted with antifade medium. Slides were stored at −20 °C in the dark, images captured with unified exposure parameters, and fluorescence intensity analyzed by ImageJ-win64 software.

#### 4.5.4. Real-Time Fluorescence Quantitative PCR

100 mg of mouse skeletal muscle tissue (specified type) was dissected on ice, snap-frozen in liquid nitrogen, and total RNA was extracted with TRIzol reagent, purified by chloroform phase separation, isopropanol precipitation and ethanol washing. RNA concentration and purity were determined by NanoDrop, with A260/280 1.8–2.1 and A260/230 > 2.0 as qualified standards. Genomic DNA removal and reverse transcription were performed with a commercial kit following the manufacturer’s protocol: gDNA removal at the recommended temperature, cDNA synthesis at 37 °C, and reaction inactivation at the specified high temperature. cDNA was stored at −20 °C for later use. Relative mRNA expression of target genes (*Nrf2, HO-1, NQO1, PGC-1α*) and reference gene (*β-actin*) was detected using SYBR Green Real-time PCR Kit (RR420A, TAKARA Bio Inc., Kusatsu, Shiga, Japan) on the ABI 7500 system. Reaction conditions: initial denaturation at 95 °C for 30 s; 40 cycles of 95 °C for 5 s and 60 °C for 34 s; melting curve analysis (95 °C 15 s, 60 °C 1 min, 95 °C 15 s) to verify primer specificity. Relative expression was calculated by the 2^−ΔΔCt^ method with 3 biological and 3 technical replicates. Primer sequences and product lengths are in Table 1.

#### 4.5.5. Western Blotting Analysis

Approximately 0.1 g of quadriceps muscle was placed into a 1.5 mL centrifuge tube along with 2 ceramic grinding beads and 1000 µL of lysis buffer (RIPA buffer + 100× protease inhibitor + 50× phosphatase inhibitor). After centrifugation at 12,000 r/min for 30 min, the supernatant was collected, mixed with 5× loading buffer, and boiled for 10 min. The sample was then cooled to room temperature and stored at −20 °C. For subsequent experiments, proteins were separated by electrophoresis using a 10% resolving gel and then transferred onto a PVDF membrane using the wet transfer method. The membrane was blocked with 5% skim milk at room temperature for 1 h. After discarding the blocking solution, the primary antibody (diluted at 1:1000) was added and incubated at 4 °C overnight. The primary antibodies used were as follows: Nrf2 antibody (R380773, Zenbio, Chengdu, China), HO-1 antibody (R24541, Zenbio), NQO1 antibody (R381695, Zenbio), PGC-1α antibody (R381615, Zenbio), NLRP3 antibody (R381207, Zenbio), IL-1β antibody (YP1000158, UppingBio, Hangzhou, China), TXNIP antibody (YP-mAb-17277, UppingBio), and β-actin antibody (4970S, Cell Signaling, Danvers, MA, USA). The following day, the membrane was warmed at room temperature for 30 min and then washed with TBST buffer three times for 15 min each. The corresponding secondary antibodies (diluted in 5% bovine serum albumin at a ratio of 1:10,000) were added based on the primary antibodies and incubated on a shaker at room temperature for 2 h. The membrane was then washed three times with TBST buffer for 15 min each. Chemiluminescent imaging was performed using a gel imaging system to analyze the protein expression of Nrf2, HO-1, and NQO1. The integrated density values of protein bands were calculated using ImageJ software. The results of Western blotting were expressed as the ratio of the integrated density values of the target protein to the reference protein.

### 4.6. Main Observation Indicators

1. The changes in related indices such as blood glucose, blood lipids, and insulin resistance in mice from each group after 6 weeks of exercise and luteolin intraperitoneal injection intervention.
2. Morphological changes in the skeletal muscle tissue of mice from each group.
3. Protein and mRNA expression of oxidative stress markers such as Nrf2, HO-1, and NQO1 in the skeletal muscle of mice from each group.
4. Protein expression levels of inflammatory factors such as NLRP3, IL-1β, and IL-18 in the skeletal muscle of mice from each group.

### 4.7. Statistical Analysis

All experimental data were analyzed using SPSS 27.0.1 and presented as mean ± standard deviation (M ± SD). One-way analysis of variance (one-way ANOVA) was performed for intergroup comparisons, followed by Tukey’s post hoc multiple comparison test. A value of *p* < 0.05 was considered statistically significant. The western blotting results were analyzed and processed by Image J software, and the histogram was drawn by GraphPad Prism 8.0 software. The statistical method of this article has been reviewed and approved by the biostatistics experts of the Medical Department of Yangtze University.

## 5. Conclusions

Collectively, aerobic exercise is closely associated with enhanced Nrf2 signaling in skeletal muscle, accompanied by upregulated antioxidant proteins (HO-1, NQO1) and reduced activation of inflammatory mediators (NLRP3, IL-1β, IL-18). These molecular alterations correlate with attenuated oxidative stress and chronic inflammation, potentially protecting skeletal muscle metabolism and ameliorating insulin resistance (IR). Notably, luteolin exerts context-dependent bidirectional effects on the Nrf2 pathway, and pharmacological restriction of exercise-induced Nrf2 activity (rather than universal inhibition) was associated with partial loss of exercise benefits, including blunted antioxidant improvements and reversed inflammatory trends, suggesting Nrf2 may be a potential mediator of exercise-induced IR protection.

This study has several limitations. First, the limited sample size necessitates future validation with expanded cohorts. Second, only one type and intensity of aerobic exercise were tested; the regulatory role of Nrf2 under different exercise conditions remains to be elucidated. Third, findings are based on correlative data and pharmacological intervention, and direct causal relationships require verification via genetic models. Addressing these limitations in future studies will facilitate the design of personalized exercise and drug treatment strategies for IR patients, optimizing therapeutic outcomes.

## Figures and Tables

**Table 1:** Sequence of gene expression primers determined by real-time PCR.

Target Genes		Genetic Sequences
*Nrf2*	Forward primer 5′-3′	AAGACTGCGTTCCTGCTCAAC
	Reverse primer 5′-3′	AAAGCCCTACAGCAACTGTCG
*HO-1*	Forward primer 5′-3′	AAGCCGAGAATGCTGAGTTCA
	Reverse primer 5′-3′	GCCGTGTAGATATGGTACAAGGA
*NQO1*	Forward primer 5′-3′	GCCGAACACAAGAAGCTGGAAG
	Reverse primer 5′-3′	GGCAAATCCTGCTACGAGCACT
*PGC-1α*	Forward primer 5′-3′	TATGGAGTGACATAGAGTGTGCT
	Reverse primer 5′-3′	CCACTTCAATCCACCCAGAAAG
*β-actin*	Forward primer 5′-3′	GCTCTGGCTCCTAGCACCAT
	Reverse primer 5′-3′	GCCACCGATCCACACAGAGT

## Data Availability

The original contributions presented in this study are included in the article. Further inquiries can be directed to the corresponding author.

## Abbreviations

The following abbreviations are used in this manuscript: .

IR	Insulin Resistance
HOMA-IR	Homeostasis model assessment-Insulin Resistance
FINS	Fasting Insulin
IRS	Insulin receptor substrate
PI3K	Phosphatidylinositol 3-Kinase
Akt	Protein Kinase B
Nrf2	Nuclear factor E2-related factor 2
ROS	Reactive Oxygen Species
HO-1	Heme Oxygenase-1
NQO1	NAD(P)H: Quinone Oxidoreductase 1
PGC-1α	Peroxisome proliferator-activated receptor gamma coactivator 1-alpha
HFD	High fat diet
TG	Triglyceride
TC	Total cholesterol
LDL-C	Low-Density Lipoprotein Cholesterol
HDL-C	High-Density Lipoprotein Cholesterol
NLRP3	NOD like receptor protein 3
IL-1β	Interleukin-1β
IL-18	Interleukin-18
TXNIP	Thioredoxin Interacting Protein
AMPK	AMP-activated protein kinase
FBG	Fasting Blood Glucose
GTT	Glucose tolerance test
